# Fcγ Receptor-Mediated Inflammation Inhibits Axon Regeneration

**DOI:** 10.1371/journal.pone.0088703

**Published:** 2014-02-11

**Authors:** Gang Zhang, Nataliia Bogdanova, Tong Gao, Julia J. Song, Mark S. Cragg, Martin J. Glennie, Kazim A. Sheikh

**Affiliations:** 1 Department of Neurology, University of Texas Medical School at Houston, Houston, Texas, United States of America; 2 Antibody and Vaccine Group, Cancer Sciences Division, Faculty of Medicine, University of Southampton, Southampton, United Kingdom; Boston Children’s Hospital and Harvard Medical School, United States of America

## Abstract

Anti-glycan/ganglioside antibodies are the most common immune effectors found in patients with Guillain-Barré Syndrome, which is a peripheral autoimmune neuropathy. We previously reported that disease-relevant anti-glycan autoantibodies inhibited axon regeneration, which echo the clinical association of these antibodies and poor recovery in Guillain-Barré Syndrome. However, the specific molecular and cellular elements involved in this antibody-mediated inhibition of axon regeneration are not previously defined. This study examined the role of Fcγ receptors and macrophages in the antibody-mediated inhibition of axon regeneration. A well characterized antibody passive transfer sciatic nerve crush and transplant models were used to study the anti-ganglioside antibody-mediated inhibition of axon regeneration in wild type and various mutant and transgenic mice with altered expression of specific Fcγ receptors and macrophage/microglia populations. Outcome measures included behavior, electrophysiology, morphometry, immunocytochemistry, quantitative real-time PCR, and western blotting. We demonstrate that the presence of autoantibodies, directed against neuronal/axonal cell surface gangliosides, in the injured mammalian peripheral nerves switch the proregenerative inflammatory environment to growth inhibitory milieu by engaging specific activating Fcγ receptors on recruited monocyte-derived macrophages to cause severe inhibition of axon regeneration. Our data demonstrate that the antibody orchestrated Fcγ receptor-mediated switch in inflammation is one mechanism underlying inhibition of axon regeneration. These findings have clinical implications for nerve repair and recovery in antibody-mediated immune neuropathies. Our results add to the complexity of axon regeneration in injured peripheral and central nervous systems as adverse effects of B cells and autoantibodies on neural injury and repair are increasingly recognized.

## Introduction

Axon regeneration is a response of injured nerve cells that is critical for the restoration of structure and function after peripheral or central nervous systems injuries; a response that is key to recovery from numerous neurological disorders. Depending on the pathophysiological situation, axon regeneration is often limited, resulting in poor recovery. Defining the molecular and cellular mechanisms that prevent regeneration of injured axons in various disease situations can provide key insights that may allow development of therapeutic approaches to enhance axon growth in neurological diseases. We present a novel mechanism involving adaptive and innate immune interactions to inhibit regeneration of injured axons with implications for a number of neuroimmunological disorders.

Guillain-Barré syndrome (GBS) is an autoimmune disorder affecting the peripheral nervous system, which is the most common cause of acute flaccid paralysis worldwide. About 20% of GBS patients are left with significant disability. Poor recovery in GBS and other neurological disorders commonly reflect failure of axon regeneration and reinnervation of targets. Anti-ganglioside/glycan antibodies (Abs) are strongly associated with the pathogenesis of GBS [1], [2]. Studies indicate that anti-gangliosides Abs in GBS patients are induced via molecular mimicry [1], [3]. Several studies have suggested that GBS patients with anti-GD1a and/or GM1 Abs are more likely to recover slowly and have poor prognosis [4]–[13]. Understanding the mechanisms underlying failure of axonal regeneration is of critical importance to devise strategies to enhance nerve repair and recovery in GBS and other immune neurological conditions.

In this context we previously examined the effects of anti-glycan Abs on peripheral nerve repair [14], [15]. We found that passive transfer of specific patient-derived or experimental anti-glycan Abs severely inhibited axon regeneration after peripheral nervous system injury [14], [15]. Overall, these observations support our hypothesis that inhibition of axon regeneration is one mechanism of poor recovery in GBS patients with anti-glycan Abs. However, the specific molecular and cellular elements of the inflammatory milieu involved in this Ab-mediated inhibition of axon regeneration are not previously defined.

In Ab-mediated inflammation, complement and/or Fcγ receptors (FcγRs) arms of innate immunity participate to produce injury. FcγRs provide an important link between the humoral and cellular immune systems to generate inflammation [16] playing vital roles in the pathogenesis of autoimmune diseases [17], [18]. Since our previous studies indicated that terminal complement complex (C5b-9) may not be relevant to Ab-mediated inhibition of axon regeneration [14], therefore, we asked whether FcγRs participate in Ab-mediated inflammation in our disease models. Here we show that anti-glycan Abs inhibit axon regeneration of injured neurons via activating FcγRs upregulated by nerve injury and macrophages recruited from the circulation are the major contributors to the inhibition of axon regeneration.

## Materials and Methods

### Ethics Statement

All studies were performed according to institutional guidelines and animals were handled according to protocols that were approved by the Animal Welfare Committee at the University of Texas Health Science Center at Houston (Protocol number: HSC-AWC-11-046) and that are in accordance with Federal guidelines. The studies using human autopsied nerve samples were approved by the Committee for the Protection of Human Subjects at the University of Texas Health Science Center at Houston (Approval number: HSC-GEN-08-0233) and it qualifies for exempt status (category#4) according to 45 CFR 46.101(b). {***CATEGORY #4*** *: Research, involving the collection or study of existing data, documents, records, pathological specimens, or diagnostic specimens, if these sources are publicly available or if the information is recorded by the investigator in such a manner that subjects cannot be identified directly or through identifiers linked to the subjects.}*

### Mice

Mutant and transgenic mice used in nerve injury models (described below), are listed in Table 1. Osteopetrotic (*op/op*), *C3*-null, *Fcer1g*-null, *Fcer1α*-null, *Fcgr2b*-null, *Fcgr3*-null were from Jackson Laboratories. Two knockout mice lines, *B4galnt1*-null and *St8sia1*-null, with altered ganglioside expression in the nervous system were bred in house. Wild type control mice (WT mice) used in the studies were littermates (such as the controls for *Fcgr4*-null, *op/op* mice, and *B4galnt1*-null, etc.) or the corresponding background matched control animals suggested and provided by the vendor (such as the controls for *Fcer1g*-null, *Fcer1α*-null, *Fcgr2b*-null, and *Fcgr3*-null, etc.).

**Table 1:** Transgenic/mutant mice used in the studies.

Strain name	Strain description
*Fcer1g*-null	lack all activating but express inhibitory FcγRIIB
*Fcer1α*-null	lack activating FcγRI only but express all other FcγRs
*Fcgr2b*-null	Lack inhibitory FcγRIIB but express all activating FcγRs
*Fcgr3*-null	lack activating FcγRIII only but express all other FcγRs
*Fcgr4*-null	lack activating FcγRIV only but express all other FcγRs
Osteopetrotic mice	mutant mice (*op/op*), devoid of colony stimulating factor 1, are macrophage and microglia deficient
*C3*-null	lack complement component C3
*B4galnt1*-null	express only simple gangliosides GM3 and GD3, but not complex gangliosides GM1, GD1a, GD1b or GT1b [50]
*St8sia1*-null	over-expresses a-series gangliosides, particularly GM1 and GD1a and do not express b-series gangliosides GD3, GD2, GD1b, and GT1b [51]

FcγRs  =  Fcγ receptors.

*Fcgr4*-null mice lack activating FcγRIV only but express all other FcγRs. Heterozygous *Fcgr4*-null mice were received from the European Conditional Mouse Mutagenesis Program consortia distributed by the Helmholtz Zentrum München, Munich, Germany. The original ES cell containing the targeting construct was developed by the international mouse knockout consortium resulting in a non-conditional deletion of the Fcgr4 gene and were initially supplied on the mixed C57BL/6;129S5/SvEvBrd background [19]. Deletion was confirmed by genomic PCR before crossing back onto the C57BL/6 background for at least 10 generations and inter-crossing to generate homozygous knock-out mice. Deletion of FcγRIV at the protein level was confirmed in the blood by flow cytometry (assessing CD11b positive monocytes) and in the spleen, liver and lymph node by immunohistochemistry (Glennie et al. unpublished observations).

### Monoclonal anti-glycan Abs

Three previously described IgG monoclonal antibodies (mAbs) against gangliosides GD1a and/or GT1b {GD1a/GT1b-2b, GD1a-1 (E6 clone), and GD1a-2b} were used in our study [20], [21].These mAbs are designated by their ganglioside specificity and IgG isotype; e.g., GD1a/GT1b-2b refers to a mAb with GD1a and GT1b specificity and IgG2b isotype. Control mouse IgGs were used as sham Abs.

### Sciatic nerve crush model

Nerve crush provides a convenient and well characterized system to study the regeneration of injured axons mimicking the regenerative response of transected/degenerating axons in GBS. Briefly, in 8 to 12-week-old age male animals sciatic nerve was crushed at mid-thigh level on one side, as described [14]. Animals were administrated various doses of specific anti-glycan mAbs or control Abs by intra-peritoneal (i.p.) route (1 mg on days 0, 3, 7, 10, and 14 after crush). Behavior test (pinprick test; twice per week) and electrophysiology studies (on day 16 post surgery) were conducted on all animals. Studies were terminated on day 17 after the crush. All animals were perfused, sciatic and tibial nerves were harvested and post-fixed in a mixture of 3% glutaraldehyde and 4% paraformaldehyde.

### Behavioral testing

To evaluate sensory functional recovery, pinprick tests were conducted on animals with sciatic nerve injury. Pin prick tests were performed 1 day prior to the surgery and on indicated days post nerve crush, as described [22]. Briefly, the lateral part of the plantar surface of the hind paw, where sciatic nerve branches sense, was divided into 5 areas. A needle was gently applied to each of those areas. It was scored 1 for this area if the animal quickly lifts, licks, or shakes its paw after the needle prick. No response was considered as 0. All testing was performed blindly.

### Electrophysiology

Sciatic nerve conduction studies were performed on all animals, as described [14]. Briefly, sciatic nerves were stimulated at sciatic notch and compound muscle action potential amplitudes were recorded in the hind-paw on day 16 post nerve crush.

### Morphometry

Sciatic nerve (∼10 mm distal to the crush site) and tibial nerve segments (∼20 mm distal to the crush site) or grafted nerve segment were embedded in Epon, as described [23]. 1µm- toluidine blue stained sections were obtained and one entire section of the whole nerve segment/animal was used for quantification, as described [15], [24]. All myelinated axons in a single whole cross section of the nerve were counted at light level (40X) by using a motorized stage and stereotactic imaging software (Axiovision; Zeiss), as described [14], [24]. This morphometric avoids random sampling and includes all regenerating nerve fibers. 12 nerves/group were used for each individual nerve crush experiment and 6 nerves/group for each nerve grafting experiment (also notated in the respective figure legends).

### Nerve graft study

The nerve graft studies were performed on age and gender matched WT control and *Fcer1g*-null mice. The sciatic nerve grafts (∼2 cm long) collected from WT or *Fcer1g*-null mice were implanted to the proximal stumps of transected sciatic nerves of host animals (WT or *Fcer1g*-null mice) with 10-0 prolene sutures. Different groups include: (1) WT nerve grafts in WT hosts; (2) *Fcer1g*-null nerve grafts in WT hosts; (3) WT nerve grafts in *Fcer1g*-null hosts; (4) *Fcer1g*-null nerve grafts in Fcer1g-null hosts. After the surgery, all animals were administered 5 doses of GD1a/GT1b-2b mAb (1mg, i.p.) The grafts were harvested 3 weeks after the surgery and middle segment of each graft was processed and embedded in Epon and morphometry was performed on 1-µm cross sections.

### Western blotting

The tissue lysates were extracted from intact and injured mouse nerves at different time points after nerve crush, or human nerves from controls and GBS patients. Protein concentration was determined and 2 µg of total protein/lane were electrophoresed, transferred to PVDF membranes, and probed with anti-Fcγ common chain [25] (US Biological) and anti-α-Tubulin or anti-β-actin (loading/internal control) (Cell Signaling) Abs.

### Immunocytochemistry (ICC)

Injured or intact mouse sciatic nerves, or human nerves from controls and GBS patients were fixed and cryoprotected. Cryosections were double labeled for Fcγ common chain (shared by all activating FcγRs) and CD68 (Macrophage marker; AbD serotech), or glial fibrillary acidic protein (GFAP) (Schwann cell marker; Santa Cruz Biotechnology), or Iba1 (microglial cell marker; Abcam) with specific Abs. The sections were then developed with specific fluorescently conjugated secondary Abs and examined by fluorescent microscopy (Zeiss).

### Human tissue harvesting and preparation

Human cauda equina, were obtained from autopsies of GBS patients (various intervals after onset of GBS, i.e., acute and post-acute phases) or patients without neuropathic disorders (controls). The human tissues were then post-fixed in 4% paraformaldehyde for 24–48 hours. Fixed tissues were cryoprotected and cryosectioned, 10–15 µm cross sections were air-dried on glass slides for immunocytochemistry studies.

### Quantitative Real-time PCR

All primer sets were from Life Technologies: 18s, Hs99999901_s1; FcγRI, Mm00438874_m1; FcγRII, Mm00438880_g1; FcγRIII, Mm00438883_m1; FcγRIV, Mm00519988_m1. Total RNA was extracted from injured or intact nerve tissues, according to manufacturer’s instruction (Invitrogen). cDNA was generated through reverse transcription from 0.2 µg of each RNA sample by using High Capacity RNA-to-cDNA master mix (Life Technologies). Real-time PCR was performed on ABI Step-One Plus (Applied Biosystems) using TaqMan Fast Advanced Master Mix (Life Technologies). 18s was used as normalization control. All PCR were conducted in triplicate and repeated at least three times.

### Ganglioside ELISA to determine kinetics of anti-glycan Abs in mice

Serum samples from WT and Mutant *op/op* mice collected at various intervals after administration of anti-glycan mAbs were used for ELISA, as described [20].

### Statistics

All numerical data are presented as mean ± s.e.m. Differences between groups were determined using Student’s *t* test or ANOVA with corrections for multiple comparisons, *p* values < 0.05 were considered statistically significant.

## Results

### Immune complex formation is required for the inhibition of axon regeneration mediated by anti-glycan Abs

**Figure 1:**
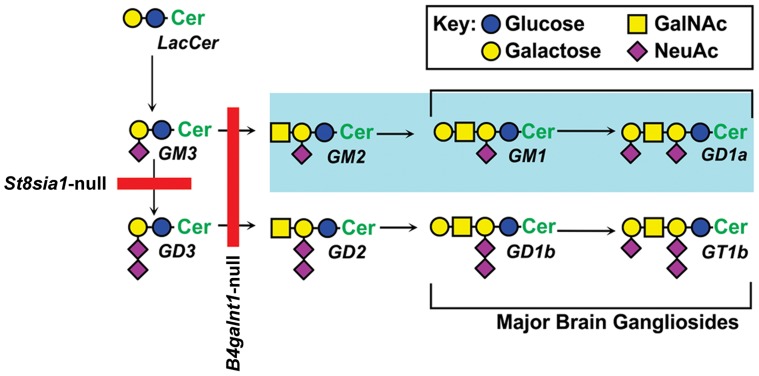
Scheme of biosynthetic pathways for major nervous system gangliosides. Blockades in ganglioside biosynthesis in knockout mice are indicated by solid red lines. GM2/GD2-synthase knockout mice (*B4galnt1*-null) mice do not express any complex gangliosides, including GM1, GD1a, GD1b or GT1b, and express only simple gangliosides GM3 and GD3. GD3-Synthase knockout mice (*St8sia1*-null), which lack the GD3 synthase required for synthesis of b-series gangliosides, overexpress a-series gangliosides (highlighted), particularly GM1 and GD1a, and do not express b-series gangliosides GD3, GD2, GD1b, and GT1b.

We examined the inhibitory effects of three GD1a-reactive mAbs in the nerve crush model because some patients with GBS and anti-GD1a Abs have poor recovery [4], [6], [9]. These mAbs were tested in WT, *B4galnt1*-null (lack all complex gangliosides), and *St8sia1*-null (lack b-series but overexpress a-series gangliosides including GD1a) (Figure 1). Our results showed that GD1a/GT1b-2b mAb induce significant inhibition of axon regeneration in WT and *St8sia1*-null animals but not in *B4galnt1*-null mice that did not express corresponding glycan antigens (Table 2), as reported previously [14]. Two anti-GD1a mAbs did not inhibit axon regeneration in WT or *B4galnt1*-null, whereas these mAbs induced severe inhibition in *St8sia1*-null mice (Table 2). These findings were consistent with our previous studies showing that higher GD1a density present in *St8sia1*-null animals {building up behind the biosynthetic block (Figure 1)} was necessary to induce anti-GD1a Ab-mediated axonal injury in mice [26]. These results emphasize the importance of immune complex formation in Ab-mediated inhibition of axon regeneration.

**Table 2:** Anti-glycan mAbs and their inhibitory effects.

Anti-glycan mAbs	Glycan specificity	Inhibition of axon regeneration		
		WT mice {No. of MF}	*St8sia1*-null mice {No. of MF}	*B4galnt1*-null mice {No. of MF}
Control Ab	None	**No** {2351±138 at SN; 576±62 at TN}	**No** {2379±227 at SN; 689±72 at TN}	**No** {1924±103 at SN; 529±42 at TN}
GD1a/GT1b-2b	GD1a/GT1b	**Yes** {354±50 (*p* < 0.05) at SN; 34±5 at TN (*p* < 0.05)}	**Yes** {254±19 (*p* < 0.05) at SN; 23±4 at TN (*p* < 0.05)}	**No** {1846±45 (*p* > 0.05) at SN; 496±14 at TN (*p* > 0.05)}
GD1a-1(E6 clone)	GD1a	**No** {2121±65 (*p* > 0.05) at SN; 535±71 at TN (*p* >0.05)}	**Yes** {792±116 (*p* < 0.05) at SN; 145±41 at TN (*p* < 0.05)}	**No** {2023±79 (*p* > 0.05) at SN; 523±30 at TN (*p* > 0.05)}
GD1a-2b	GD1a	**No** {2062±52 (*p* >0.05) at SN; 566±35 at TN (*p* >0.05)}	**Yes** {432±66 (*p* < 0.05) at SN; 48±8 at TN (*p* < 0.05)}	**No** {1937±101 (*p* > 0.05) at SN; 536±35 at TN (*p* > 0.05)}

The table contains the name of all anti-glycan mAbs used in the study, their glycan specificity, and their inhibitory effects on axon regeneration in WT and transgenic mice with altered ganglioside expression. Comparisons were made between control and anti-ganglioside Ab treated-animals, and the differences were determined using Student’s t-test. *p* < 0.05 was considered as significant. WT  =  wild type; MF  =  myelinated nerve fiber; SN  =  sciatic nerve; TN  =  tibial nerve; mAbs  =  monoclonal antibodies.

### FcγRs are up-regulated in injured nerves of mice and GBS patients

**Figure 2:**
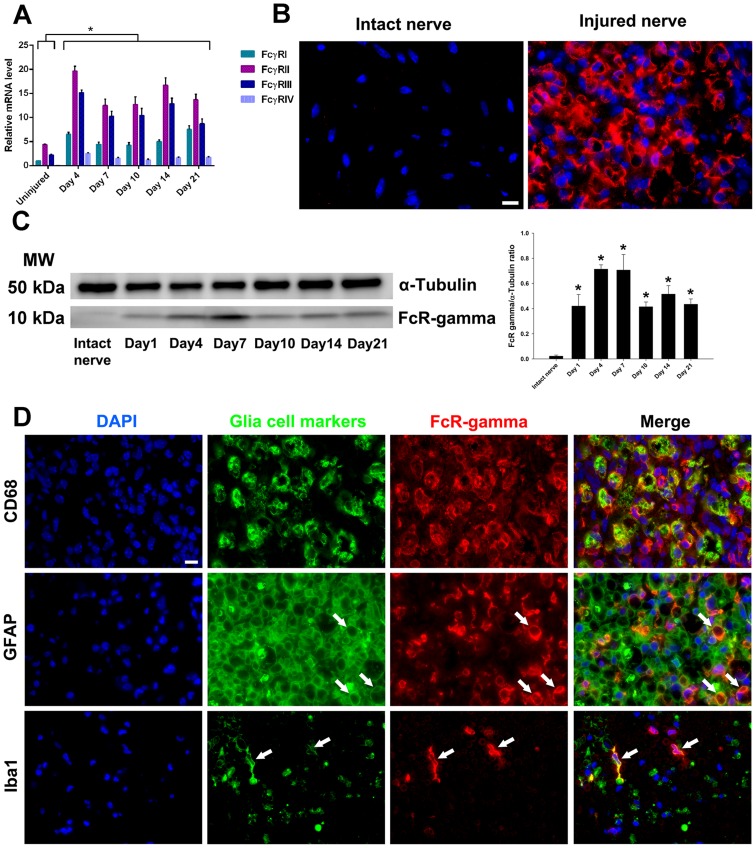
Upregulation of Fcγ receptors expression in injured mouse nerves. Quantitative PCR showing the relative mRNA levels of FcγRI, FcγRII, FcγRIII, and FcγRIV in injured nerves at various time points after surgery (A). Single labeling immunocytochemistry studies showing the expression of Fcγ common chain (shared by all activating Fcγ receptors) in the injured sciatic nerves (B). Western blotting images and quantitative densitometry of Fcγ common chain in the injured nerves (C). Double labeling immunocytochemistry studies showing the activating Fcγ receptors by endogenous {microglia (Iba1 positive; 1 day after injury) and Schwann cells (GFAP positive; 4 days after injury} and recruited {macrophages (CD68 positive); 14 days after injury} glia in the injured nerves (D). *p < 0.001 (One-way ANOVA, Bonferroni’s’ post-hoc test). Arrows showing the co-localization between activating Fcγ receptors signal (Fcγ common chain) and glia cell markers (GFAP and Iba1). N  =  4–5 per group. Error bars, s.e.m. Scale bar, 10 µm.

First, we examined the expression of FcγRs in mouse nerve crush model and compared it to GBS tissues. Quantitative PCR studies showed highly significant upregulation (range 3–20 fold) of different FcγRs (activating and inhibitory) at mRNA level in injured nerves compared to uninjured control nerves. mRNA of FcγRII and FcγRIII had comparatively higher expression levels throughout the study period compared to other FcγRs (Figure 2A). Upregulation of activating FcγRs was confirmed at protein level by ICC and immunoblotting on uninjured and injured nerve segments. FcγRs were undetectable in uninjured nerves and their expression was significantly upregulated in injured nerves (Figures 2B and 2C). There are two macrophage populations, namely resident and hematogenous monocyte-derived macrophages, which respond and participate in degenerative and regenerative responses after mammalian neural injury [27]. Reliable immunologic and phenotypic markers that can differentiate between the two macrophage populations are not readily available. However, resident macrophages such as microglia usually act as an early responder to the nerve injury, prior to the massive influx of hematogenous macrophages [28] and temporal profiling allows distinguishing these populations. The ICC studies showed that activating FcγRs were expressed by resident (Schwann and microglial cells) and recruited (macrophages) glia in injured nerves (Figure 2D). The upregulation of activating FcγRs on resident glia including microglia and Schwann cells was confirmed at time points prior to the significant recruitment of circulating macrophages in injured nerves.

**Figure 3:**
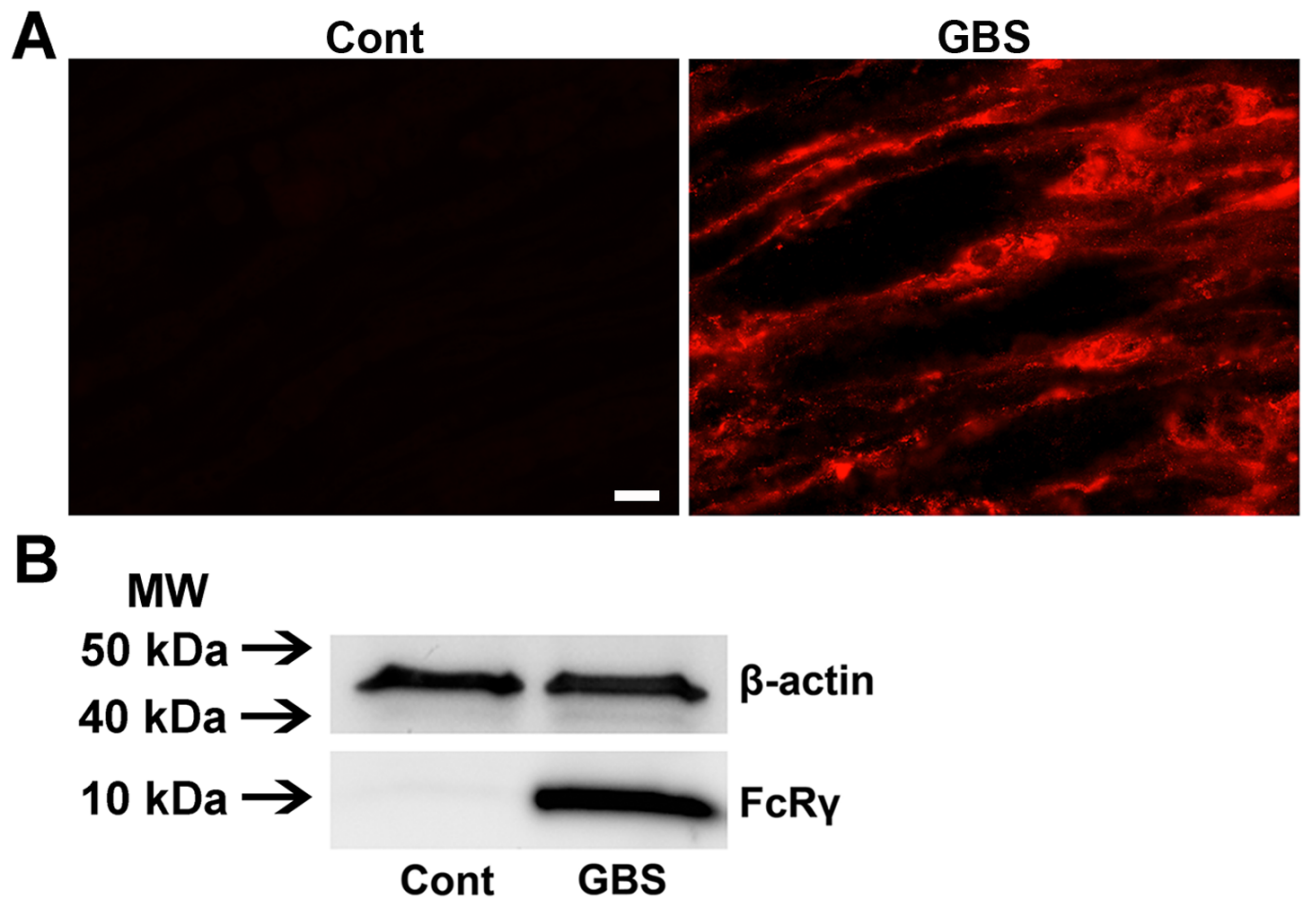
Upregulation of Fcγ receptors in nerves of GBS patients. Micrographs showing strong signal of Fcγ common chain in the nerve of a GBS patient but not in a control nerve (A). Western blotting images showing significant upregulation of Fcγ common chain expression in GBS nerves compared to controls (B). Scale bar, 10 µm.

Examination of GBS nerves, obtained at different time points after onset, showed that there was significant upregulation of Fcγ common chain in acute (data not shown) and post-acute phase of GBS nerves, whereas control/uninjured human nerves did not show staining for Fcγ common chain (Figure 3A). Western blotting studies confirmed ICC findings (Figure 3B). These results provide evidence that activating FcγRs are upregulated in GBS nerves and injured nerves of experimental animals and are available to participate in Ab-mediated inflammation.

### Activating FcγRs are required for the inhibition of axon regeneration mediated by anti-glycan Abs

**Figure 4:**
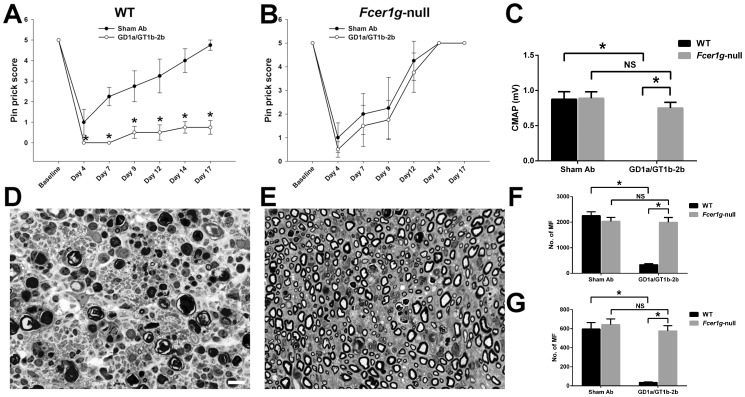
Anti-glycan Ab-mediated inhibition of axon regeneration was dependent on the expression of activating FcγRs. Behavioral studies (Pinprick test) showing the effects of GD1a/GT1b-2b mAb and sham Abs on sensory functional recovery after nerve injury in WT control mice (A), or *Fcer1g*-null mice (B). *p < 0.01 (Student’s *t* test). Quantitative electrophysiology showing the CMAP amplitudes of GD1a/GT1b-2b mAb- or sham Ab-treated WT or *Fcer1g*-null mice (C). Representative micrographs from sciatic nerve segments distal to the crush site in GD1a/GT1b-2b mAb-treated WT (D) or *Fcer1g*-null (E) mice. Morphometric analysis (MF counts) in sciatic (F) or tibial (G) nerves from WT or *Fcer1g*-null mice treated with sham Ab or GD1a/GT1b-2b mAb. *p < 0.001 (Two-way ANOVA, Tukey’s multiple comparisons test). N  =  10–12 per group. Error bars, s.e.m. Scale bar, 10 µm. WT  =  wild type; NS  =  not significant; MF  =  myelinated nerve fibers.

We tested the inhibitory effects of GD1a/GT1b-2b in nerve crush model on axon regeneration in *Fcer1g*-null mice, which lack all activating FcγRs and only express inhibitory FcγRIIB [29]. Our results showed that *Fcer1g*-null mice were resistant to the severe inhibitory effects seen in background-matched WT mice, as assessed by behavioral, electrophysiological, and morphometric measures. Behavioral studies (Pinprick test) showed that GD1a/GT1b-2b mAb significantly reduced sensory functional recovery after nerve injury in WT mice compared to WT animals treated with sham Abs (Figure 4A), whereas the sensory functional recovery was similar in *Fcer1g*-null mice treated with GD1a/GT1b-2b and sham Abs (Figure 4B). Quantitative electrophysiology (sciatic nerve conductions) indicated that GD1a/GT1b-2b mAb adversely affected the motor nerve regeneration and target (muscle) reinnervation in WT animals but not in *Fcer1g*-null mice (Figure 4C). Morphological studies also demonstrated that the GD1a/GT1b-2b mediated inhibition of axon regeneration found in WT mice was reversed in *Fcer1g*-null mice (Figures 4D-4G). There was significant decrease in regenerating myelinated nerve fibers (MF) in GD1a/GT1b-2b-treated WT animals at sciatic (SN) (336±38) and tibial (TN) (36±5) nerves compared with sham Ab-treated WT mice in sciatic (2253±152) and tibial (596±69) nerves (Figures 4D, 4F, and 4G). In contrast, no significant difference in MF regeneration was found in *Fcer1g*-null mice treated with GD1a/GT1b-2b (SN, 1995±187 and TN, 577±55) or sham Ab (SN, 2032±156 and TN, 642±60) (Figures 4E, 4F, and 4G).

### Inhibitory FcγRIIB is not involved in the anti-glycan Abs-mediated inhibition of axon regeneration

**Figure 5:**
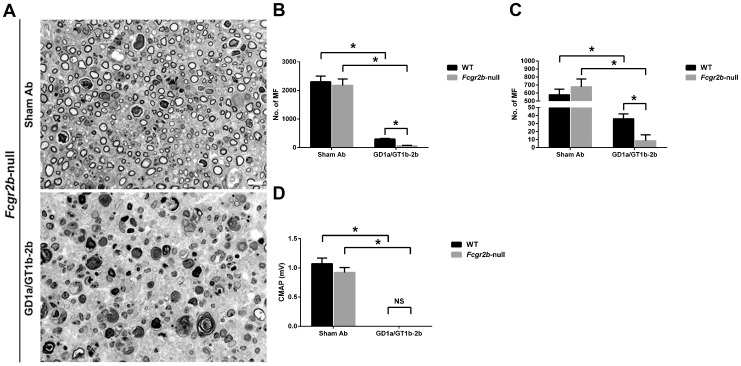
Anti-glycan Ab-mediated inhibition of axon regeneration was independent of inhibitory FcγRIIB. Sciatic nerve micrographs from *Fcgr2b-null* mice treated with sham Ab or GD1a/GT1b-2b mAb (A). MF counts in sciatic (B) or tibial (C) nerves from WT or *Fcgr2b*-null mice treated with sham Ab or GD1a/GT1b-2b mAb. Quantitative electrophysiological data showing CMAP amplitudes in WT mice or *Fcgr2b*-null mutants treated with sham Ab or GD1a/GT1b-2b mAb (D). *p < 0.001 (Two-way ANOVA, Tukey’s multiple comparisons test). N  =  12 per group. Error bars, s.e.m. Scale bar, 10 µm. WT  =  wild type; NS  =  not significant; MF  =  myelinated nerve fibers.

The inhibition of axon regeneration induced by GD1a/GT1b-2b was abrogated in *Fcer1g*-null mice, which exclusively express inhibitory FcγRIIB. This led to the hypothesis that the inhibitory FcγRs are not involved in the anti-glycan Ab-mediated inhibition. Availability of *Fcgr2b* -null mice (which lack inhibitory FcγRIIB but express all activating FcγRs) allowed reconstitution studies. We found that axon regeneration in *Fcgr2b* -null mice was severely inhibited by GD1a/GT1b-2b mAb. The numbers of regenerating MF in sciatic (57±19) and tibial (9±8) nerves in GD1a/GT1b-2b-treated *Fcgr2b* -null mice were significantly decreased compared with those in sham Ab-treated sciatic (2177±225) and tibial (678±97) nerves (Figures 5A-5C). GD1a/GT1b-2b induced even more severe inhibition of axonal regeneration in *Fcgr2b* -null mice (SN, 57±19 and TN, 9±8) than what it did in the control animals (SN, 299±22 and TN, 36±6). Electrophysiological studies were consistent with morphological findings showing that the treatment with GD1a/GT1b-2b mAb abolished evoked CMAP responses in *Fcgr2b -null* mutants (Figure 5D). These studies in *Fcer1g*-null and *Fcgr2b* -null mice indicated that anti-glycan Ab-mediated inhibition of axon regeneration in this model was completely dependent on the expression of activating FcγRs.

### Activating FcγRIII, but not FcγRI and FcγRIV, is involved in the anti-glycan Ab-mediated inhibition of axon regeneration

Activating FcγRs include FcγRI, FcγRIII, and FcγRIV, which differ in their Ab binding affinities and isotype specificity due to their different molecular structures [30]. In order to evaluate the role of different individual activating FcγRs in the Ab-mediated axonal inhibition, the inhibitory effect of GD1a/GT1b-2b was examined in three different transgenic lines *Fcer1α*-null, *Fcgr3*-null, and *Fcgr4*-null, which lack activating FcγRI, FcγRIII, or FcγRIV, respectively.

**Figure 6:**
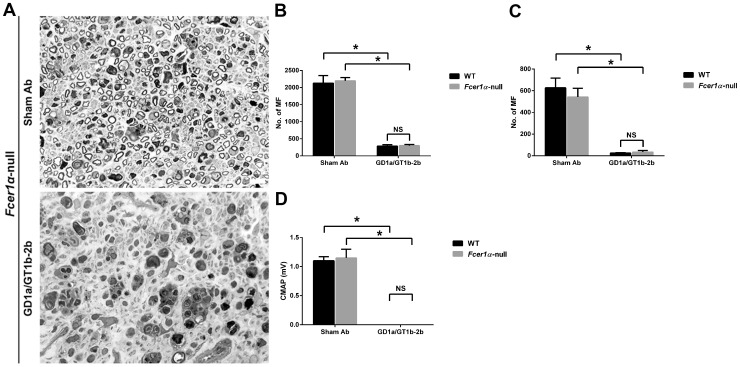
Anti-glycan Ab-mediated inhibition of axon regeneration was independent of activating FcγRI. Sciatic nerve micrographs from *Fcer1α*-null mice treated with sham Ab or GD1a/GT1b-2b mAb (A). MF counts in sciatic (B) or tibial (C) nerves from WT or *Fcer1α*-null mice treated with sham Ab or GD1a/GT1b-2b mAb. Quantitative electrophysiological data showing CMAP amplitudes in WT or *Fcer1α*-null mice treated with sham Ab or GD1a/GT1b-2b mAb (D). *p < 0.001 (Two-way ANOVA, Tukey’s multiple comparisons test). N  =  12 per group. Error bars, s.e.m. Scale bar, 10 µm. WT  =  wild type; NS  =  not significant; MF  =  myelinated nerve fibers.

**Figure 7:**
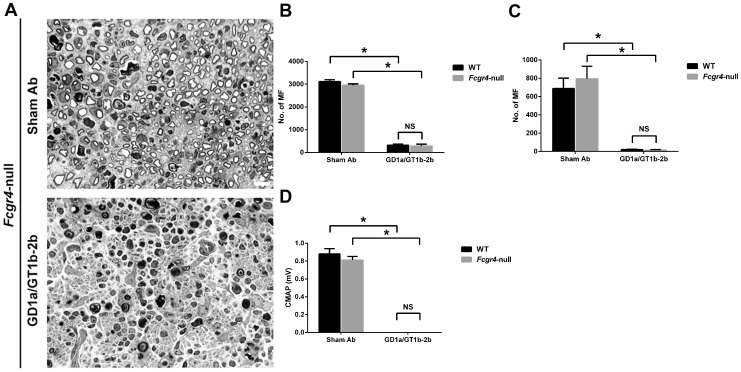
Anti-glycan Ab-mediated inhibition of axon regeneration was independent of activating FcγRIV. Sciatic nerve micrographs from *Fcgr4*-null mice treated with sham Ab or GD1a/GT1b-2b mAb (A). MF counts in sciatic (B) or tibial (C) nerves from WT or *Fcgr4*-null mice treated with sham Ab or GD1a/GT1b-2b mAb. Quantitative electrophysiological data showing CMAP amplitudes of WT or *Fcgr4*-null mice treated with sham Ab or GD1a/GT1b-2b mAb (D). *p < 0.001 (Two-way ANOVA, Tukey’s multiple comparisons test). N  =  12 per group. Error bars, s.e.m. Scale bar, 10 µm. WT  =  wild type; NS  =  not significant; MF  =  myelinated nerve fibers.

**Figure 8:**
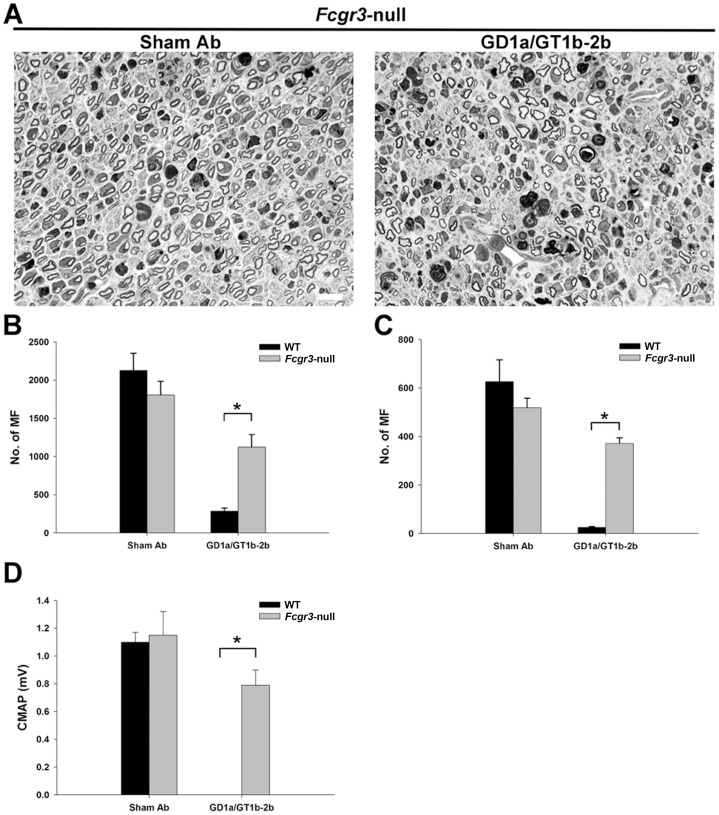
Anti-glycan Ab-mediated inhibition of axon regeneration was dependent on the expression of activating FcγRIII. Sciatic nerve micrographs from *Fcgr3*-null mice treated with GD1a/GT1b-2b and sham Abs (A), notably, *Fcgr3*-null mice treated with GD1a/GT1b-2b have many more regenerating MF compared to WT animals treated with the same Ab (compare Figure 4D). Morphometry showing MF counts in WT mice and *Fcgr3*-null mice treated with sham Ab or GD1a/GT1b-2b mAb at sciatic (B) and tibial (C) nerve levels. Electrophysiology showing CMAP amplitudes of WT or *Fcgr3-*null mutants treated with sham Ab or GD1a/GT1b-2b mAb (D). *p < 0.01 (Student’s *t*-test). N  =  12 per group. Error bars, s.e.m. Scale bar, 10 µm. WT  =  wild type; MF  =  myelinated nerve fibers.

Our results showed that *Fcer1α*-null and *Fcgr4*-null mice were susceptible to anti-glycan Ab-mediated inhibition of axon regeneration (Figures. 6 and 7), whereas this inhibitory effect was almost completely reversed in *Fcgr3*-null mice (Figure 8). We found that anti-glycan Ab-mediated inhibition of axon regeneration was independent of activating FcγRI. The morphological data showed that the treatment with GD1a/GT1b-2b significantly reduced the numbers of regenerating MF in sciatic (298±35) and tibial (34±14) nerves in *Fcer1α*-null mice compared with sham Ab treatment (2197±93 at sciatic and 542±80 at tibial nerve levels) (Figures 6A-6C). There was no significant difference in anti-glycan Ab induced inhibition of axon regeneration between *Fcer1α*-null mice (SN, 298±35 and TN, 34±14) and WT control animals (SN, 286±39 and TN, 25±3). Electrophysiological studies were consistent with morphological findings showing that the treatment with GD1a/GT1b-2b mAb abolished evoked CMAP responses in *Fcer1α*-null mutants (Figure 6D). Next, our studies indicated that anti-glycan Ab-mediated inhibition of axon regeneration was also independent of activating FcγRIV. We found that there was a significant decrease in numbers of regenerating MF in GD1a/GT1b-2b-treated sciatic (282±82) and tibial (15±3) nerves in *Fcgr4*-null mice compared with sham Ab treated sciatic (2952±64) and tibial (795±68) nerves (Figures 7A-7C). The anti-glycan Ab mediated inhibition of axon regeneration was similar between *Fcgr4*-null mice (SN, 282±82 and TN, 15±3) and their littermate control animals (SN, 320±50 and TN, 21±2). Electrophysiological studies were consistent with morphometry showing that the treatment with GD1a/GT1b-2b mAb abolished evoked CMAP responses in *Fcgr4*-null mutants (Figure 7D).

In contrast to *Fcer1α*- and *Fcgr4*-null mice, we found that FcγRIII-null mice were resistant to anti-glycan Ab-mediated inhibition of axon regeneration. Morphometry showed that GD1a/GT1b-2b-induced inhibition of axon regeneration observed in WT was largely reversed in *Fcgr3*-null mice at sciatic (Figures 8A and 8B) and tibial (Figure 8C) nerve levels. In this set of studies, number of regenerating MF in WT mice treated with GD1a/GT1b-2b were 286±39 at sciatic and 25±3 at tibial nerve levels compared to sham Ab-treated group (SN, 2128±223; TN 627±89). In comparison, number of regenerating MF in GD1a/GT1b-2b-treated *Fcgr3 -null* mice were1125±162 at sciatic and 371±23 at tibial nerve levels, whereas number of regenerating MF in sham Ab-treated *Fcgr3 -null* mice were 1807±178 at sciatic and 519±39 at tibial nerve levels. Electrophysiology showed that *Fcgr3 -null* mutants treated with GD1a/GT1b-2b mAb had significant recovery of evoked CMAP amplitudes compared to WT mice treated with GD1a/GT1b-2b mAb, thus, confirming the functional recovery of regenerating fibers (Figure 8D). Overall, these studies indicate that activating FcγRIII is the dominant FcγR involved in GD1a/GT1b-2b mAb-mediated inhibition.

### Macrophage and microglial populations are involved in Ab-mediated inhibition of axon regeneration

*Op/op* mice with macrophage and microglia deficiency were examined to determine whether anti-glycan Abs mediated inhibition of axon regeneration depends on FcγRs-bearing macrophage/microglial population. Inhibitory effects of GD1a/GT1b-2b mAb on axon regeneration were compared in *op/op* mice and WT littermates in nerve crush model.

**Figure 9:**
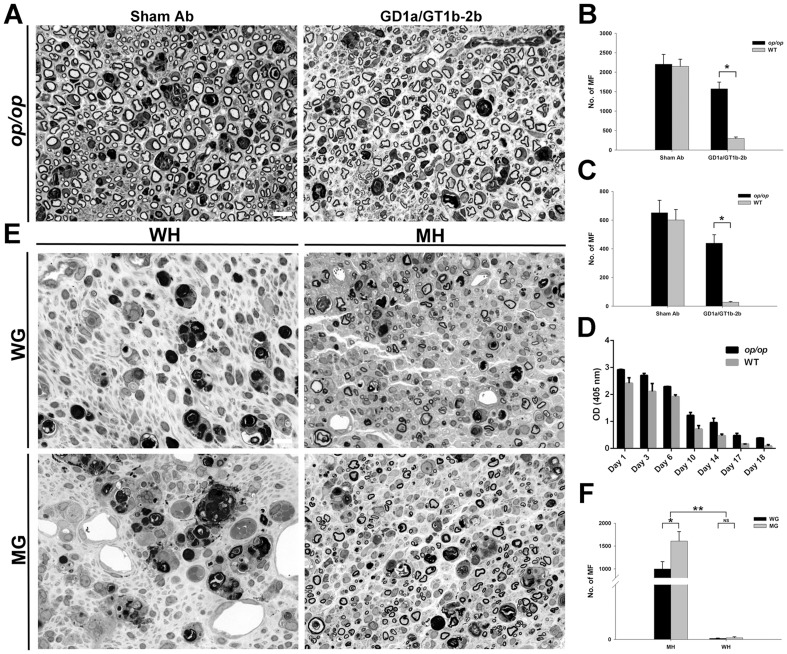
Activating FcγRs-bearing macrophages were major contributors to inhibition of axon regeneration. Sciatic nerves showing regeneration of MF in *op/op* mice treated with sham or GD1a/GT1b-2b mAb (A). Morphometry data showing that number of regenerating MF in WT mice and *op/op* mice treated with sham Ab or GD1a/GT1b-2b at sciatic (B) and tibial (C) nerve levels. *p < 0.01 (Student’s *t*-test). N  =  12 per group. Serology showing the levels of circulating GD1a/GT1b-2b mAb in *op/op* mice and WT animals (D). Micrographs (E) and morphometry (F) showing that mutant graft transplanted in mutant host had the most regeneration, wild type graft transplanted in mutant host had some regenerating fibers, and wild type graft or mutant graft transplanted in wild type hosts had virtually no regenerating fibers. *p < 0.05; **p < 0.001 (Two-way ANOVA, Bonferroni’s post-hoc test). N  =  6 per group. Error bars, s.e.m. Scale bar, 10 µm. WG  =  wild type grafts, WH  =  wild type hosts, MG  =  mutant (*Fcer1g-null*) grafts, and MH  =  mutant (*Fcer1g-null*) hosts. NS  =  not significant; MF  =  myelinated nerve fibers.

Our results showed that *op/op* mice had significantly more regenerated axons compared to WT animals indicating the involvement of macrophage/microglial cells in the anti-glycan Ab-mediated inhibition (Figures 9A-9C). Morphometry showed that number of regenerating MF in *op/op* mice treated with GD1a/GT1b-2b was significantly higher than WT mice treated with the same mAb at sciatic (Figure 9B) and tibial (Figure 9C) nerve levels. Quantification of MF fiber showed that WT mice treated with sham Abs had 2151±181 at sciatic and 601±72 at tibial nerve levels, whereas WT mice treated with GD1a/GT1b-2b had 299±37 at sciatic and 27±5 at tibial nerve levels; *op/op* mice treated with sham Abs had 2203±252 at sciatic and 651±87 at tibial nerve levels. In comparison, *op/op* mice treated with GD1a/GT1b-2b had 1572±171 at sciatic and 439±59 at tibial nerve levels. Serological studies showed that the levels of circulating GD1a/GT1b-2b mAb in *op/op* mice were comparable to their WT counterparts (Figure 9D), demonstrating that IgG homeostasis was not altered in these mice.

### Recruited macrophages are the major contributor to the Ab- and activating FcγRs-mediated inhibition of axon regeneration

Our results showed that both endoneurial glia (Schwann and microglial cells) and recruited macrophages express activating FcγRs (Figure 2D), therefore, we asked which FcγRs-expressing cells participate in producing an inflammatory inhibitory milieu in the injured nerve.

A nerve grafting paradigm, in which nerve segments of donor mice (WT or *Fcer1g*-null/ activating FcγRs-null) were transplanted into host animals (WT mice or *Fcer1g*-null), that allows determination of the contribution of circulating macrophages (recruited in injured nerves from hosts) and resident endoneurial glial cells (in grafted nerve segments from donors) in Ab-mediated inhibition of axon regeneration was used to address this question. Previous studies had established that grafted nerve segments retain donor glia [31], [32]. These chimeric animals were administered GD1a/GT1b-2b Ab and axon regeneration was assessed in the grafted nerve segments. We found that *Fcer1g*-null hosts implanted with nerve grafts from *Fcer1g*-null donors were not susceptible to Ab-mediated inhibition, whereas WT hosts implanted with WT nerve grafts showed severe inhibition (Figures 9E and 9F). Notably, the axon regeneration in WT or *Fcer1g*-null grafts implanted in *Fcer1g*-null hosts was more pronounced compared to WT or *Fcer1g*-null grafts implanted in WT hosts. Additionally, WT grafts implanted in *Fcer1g*-null hosts had modest but significant reduction in number of regenerating axons compared to *Fcer1g*-null grafts implanted in *Fcer1g*-null hosts (Figures 9E and 9F). Morphometry showed the numbers of MF in different chimeras is as follows: 1) *Fcer1g-null* grafts in mutant *Fcer1g-null* hosts had 1609±206 MF; 2) WT grafts in *Fcer1g-null* hosts had 996±162 MF; 3) WT grafts in WT hosts had 4±1 MF; and 4) *Fcer1g-null* grafts in WT hosts had 6±4 MF.

Overall, these studies showed that the macrophages recruited from the circulation of the host animals were the dominant cell type and endogenous nerve glia had minor contribution to the activating FcγRs-induced inflammation and Ab-mediated inhibition of axon regeneration.

### Complement activation is not involved in anti-glycan Ab induced inhibition of axon regeneration

**Figure 10:**
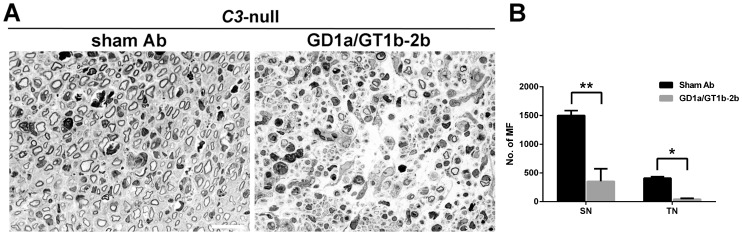
Anti-glycan Abs induced inhibition of axon regeneration in *C3*-null mice. Light micrographs of sciatic nerve from sham Ab- or GD1a/GT1b-2b-treated *C3*-null mice (A). Regenerating MF counts in sciatic and tibial nerves from *C3*-null mice treated with sham Ab or GD1a/GT1b-2b mAb (B). *p<0.05; **p<0.001 (Student’s *t*-test). N  =  12 per group. Error bars, s.e.m. Scale bar, 10 µm. MF  =  myelinated nerve fibers.

Complement-fixing pathogenic Abs can cause nerve injury through the activation of classical complement pathway. We previously demonstrated that membrane attack complex (C5b-9) is not involved in anti-glycan Ab-induced inhibition of axon regeneration [14]. In this study we examined the role of C3 complement component, a critical component of classical complement pathway, in Ab-mediated inhibition. The effect of anti-glycan mAb, GD1a/GT1b-2b, was examined in *C3*-null mice. We found that GD1a/GT1b-2b mAb produced severe inhibition of axon regeneration in *C3*-null mice (Figure 10). The numbers of regenerating MF in GD1a/GT1b-2b-treated sciatic (354±90) and tibial (42±9) nerves were significantly reduced compared with that in sham Ab treated sciatic (1501±84) and tibial (409±25) nerves. These data provide further evidence that complement is not involved in this Ab-mediated inhibition of axon regeneration in our models.

## Discussion

After peripheral nerve injury, myelin and associated myelin inhibitors (endogenous inhibitors) are rapidly cleared out. This usually makes room for the regeneration of injured peripheral nerves. However the poor prognosis and/or incomplete recovery found in some patients with monophasic autoimmune neurological disorders, such as GBS, implicate that besides these known endogenous factors, there could be exogenous inhibitors of axon regeneration in the milieu of injured nerves. We previously linked anti-glycan Abs with the inhibition of axon regeneration in an animal model of nerve repair [14], [15]. In the current study, we showed that Ab-mediated inhibition was attributable to macrophage-mediated inflammation, and these detrimental effects of macrophage-mediated inflammation were context-dependent and the presence of disease relevant anti-neural autoantibodies in the milieu of injured nerves steered the macrophage responses towards growth inhibition via specific activating FcγRs. These results indicate that the Fab fragments of anti-glycan Abs engage specific gangliosides on the tips of injured axons to form immune complexes (ICs) and the Fc portions of the same Abs interact with FcγRIII on glia/macrophages recruited in the injured nerves to induce inflammation that is hostile to regenerating axons.

We found that the formation of ICs was required for the anti-glycan Ab-mediated inhibition of axonal regeneration, as reported previously [14]. Studies with GD1a/GT1b-2b in mutant mice lacking individual activating FcγR showed that FcγRIII was the dominant receptor involved in inhibition of axon regeneration. To initiate inflammation, IgG Abs/ICs bind FcγRs, which are classically described as activating or inhibitory FcγRs, signaling through immunoreceptor tyrosine activation motifs or immunoreceptor tyrosine inhibitory motifs, respectively. The relative affinity of IgG Fc for respective FcγRs, as well as the expression levels of activating and inhibitory FcγRs, dictates the ensuing inflammatory response, which has been reviewed extensively [16], [17], [33]. In mice, the members of FcγR family include activating FcγRI, FcγRIII, and FcγRIV and inhibitory FcγRIIB receptors; homologous FcγRs are found in other mammals including humans.

The role of FcγRs and macrophage has not been previously examined in experimental studies examining the pathogenic effects of anti-ganglioside Abs on peripheral nerves. The current study directly links an IgG2b mAb (GD1a/GT1b-2b) with low affinity activating FcγRIII. FcγRIV-null mice showed GD1a/GT1b-2b-mediated severe inhibition similar to WT mice. This is surprising to us as mouse IgG2b Abs have been reported to have higher affinity for FcγRIV compared to FcγRIII and ICs formed by this isotype preferentially bind FcγRIV in mouse models of inflammation [34]. We found FcγRI was not involved in Ab-mediated injury and among the activating FcγRs, FcγRI has the highest Ab binding affinity, and it is the only FcγR, which can bind monomeric IgGs. The remaining activating FcγRs have lower binding affinities and only bind ICs [30]. We also showed that inhibitory FcγRIIB were not directly involved in Ab-mediated inhibition of axon regeneration. However, FcγRIIB could negatively regulate activating FcγRs and resultant inflammation. We believe that exaggerated inflammation in FcγRIIB-null mice could be the basis of more severe GD1a/GT1b-2b Ab-mediated inhibition of axon regeneration. GD1a-1 (E6 clone), an IgG1 mAb, induced inhibition in *St8sia1*-null (mice that overexpress GD1a) animals also supports the pathogenic role of activating FcγRIII in our model as IgG1 isotype is known to bind only FcγRIII among activating FcγRs [30]. The molecular mechanisms downstream of ICs binding to activating FcγRs and leading to proinflammatory response and associated inhibition of axon regeneration in injured nerves are not ascertained in this study. However, our previous morphological studies in this model favor that proinflammatory mediators secreted by immune effector cells, such as cytokines and chemokines, adversely influenced axon regeneration because the ultrastructure of injured axonal tips was consistent with dystrophic inhibited growth cones (reminiscent of dystrophic bulbs originally described by Ramon y Cajal [35]) and there was no attempt to phagocytose these aberrant structures [14]. This pathological change can also be seen in GBS patients with poor recovery [13].

Our studies demonstrated that monocyte-derived macrophages recruited from the circulation were the dominant cells responsible for the anti-glycan Ab-mediated inhibition of axon regeneration. We found that FcγRs are significantly upregulated in injured nerves and protein level detection was only possible after injury, consistent with previous reports [36], [37]. Our data showed that the elevated expression of FcγRs was not restricted to macrophages but also expressed by the resident glia (microglia and Schwann cells) after peripheral nerve injury. Studies in *op/op* mice and nerve grafting experiments in WT and *Fcer1g*-null mice determined that recruited macrophages played dominant role in the Ab-mediated inhibitory effect. Resident glia are also minor contributors to Ab-mediated inhibition of axon regeneration was supported by the studies showing WT nerve grafts in *Fcer1g*-null hosts had significantly less regenerating fibers compared to *Fcer1g*-null nerve grafts in *Fcer1g*-null hosts (Figures 9E and 9F). Our experimental strategy does not allow to distinguish between the contributions of Schwann cell and microglia to this ‘minor’ inhibitory component.

The present study confirmed that C3 complement component was not involved in the anti-glycan Ab-mediated deleterious effects on repair of injured nerve fibers. This finding in conjunction with our previous results showing that C5 deficient animals are susceptible to anti-glycan Ab-mediated inhibition of axon regeneration [14] indicate that complement-induced inflammation is not directly involved in our animal model. We examined complement arm of innate immunity because this has been implicated in experimental models of anti-glycan Ab-mediated injury to intact nerve fibers [38]–[41]. These studies demonstrate the participation of classical pathway including activation of C3 complement component and terminal complement complex (C5b-9). In addition to complement, whether or not FcγRs are involved in anti-glycan Ab-mediated injury to intact nerve fibers has not been examined previously.

Our results provide one potential explanation for poor recovery in GBS and indicate that immune effectors, such as anti-neural Abs, exogenous to the nervous system, can not only injure intact nervous system in acute phase of the disease but can also substantially impair the neural repair during recovery phase. Most of the previous work in the context of autoantibody-associated neuroimmunological disorders has focused on identifying immune mechanisms involved in injury to the intact nervous system, whereas our results emphasize the pathogenic effects of anti-neural Abs on nerve repair. Identification of activating FcγRs as mediators of Ab-induced inhibition of axon regeneration is relevant to the clinical observations showing that certain polymorphisms in activating FcγR genes correlated with risk of developing GBS, severity of disease, and prognosis [42], [43]. Our findings on GBS patient nerves provide evidence that activating FcγRs are upregulated on macrophages in acute and post-acute phase and can potentially participate in Ab-mediated inhibition of axon regeneration.

Collectively, we demonstrate that specific anti-glycan Abs induced inhibition of axon regeneration by triggering neuroinflammation via engagement of specific activating FcγRs on glial cells and converted proregenerative environment of acutely injured mammalian peripheral nerves to an inhibitory milieu for axon regeneration. Our studies identify molecular and cellular components of inflammatory cascade that adversely modulate axon repair. These findings support the notion that macrophage-mediated inflammation could be beneficial or harmful depending upon the presence or absence of other cues in the environment of injured nervous system. This inflammatory cascade of inhibition of axon regeneration may be under recognized and could have wider biological implications as naturally occurring anti-neural Abs and those generated secondary to neural injury (including anti-glycan Abs) are seen in a variety of settings [6], [44]–[49] including chronic immune neuropathies, multiple sclerosis, and traumatic spinal cord and brain injuries in which axonal damage and failure of axon regeneration are central to poor recovery.
